# 2,5-Dichloro­anilinium 4-chloro­benzene­sulfonate

**DOI:** 10.1107/S1600536811010518

**Published:** 2011-03-26

**Authors:** K. Shakuntala, Sabine Foro, B. Thimme Gowda

**Affiliations:** a Department of Chemistry, Mangalore University, Mangalagangotri 574 199, Mangalore, India; b Institute of Materials Science, Darmstadt University of Technology, Petersenstrasse 23, D-64287 Darmstadt, Germany

## Abstract

In the crystal of the title compound, C_6_H_6_Cl_2_N^+^·C_6_H_4_ClO_3_S^−^, the 2,5-dichloroanilinium cations and 4-chlorobenzenesulfonate anions are located on a crystallographic mirror plane and are connected by N—H⋯O hydrogen bonds. In the crystal, the connectivity of the hydrogen bonds leads to double chains propagating in [010].

## Related literature

For the effect of substituents on the oxidative strengths of *N*-chloro, *N*-aryl­sulfonamides, see: Gowda *et al.* (2004*a* ▶). For their effect on the structures of *N*-(ar­yl)-amides, see: Gowda *et al.* (2004*b* ▶) and of *N*-(ar­yl)-methane­sulfonamides, see: Gowda *et al.* (2007 ▶).
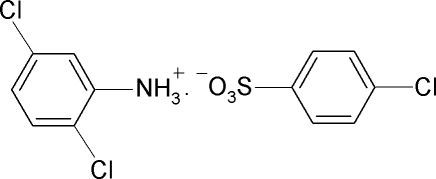


## Experimental

### 

#### Crystal data

C_6_H_6_Cl_2_N^+^·C_6_H_4_ClO_3_S^−^
*M* *_r_* = 354.62
Monoclinic, 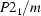

*a* = 9.792 (1) Å
*b* = 6.802 (1) Å
*c* = 10.879 (1) Å
β = 94.26 (1)°
*V* = 722.60 (15) Å^3^
*Z* = 2
Mo *K*α radiation
μ = 0.78 mm^−1^
*T* = 293 K
0.40 × 0.34 × 0.24 mm

#### Data collection

Oxford Diffraction Xcalibur diffractometer with a Sapphire CCD detector
Absorption correction: multi-scan (*CrysAlis RED*; Oxford Diffraction, 2009 ▶) *T*_min_ = 0.745, *T*_max_ = 0.834
2700 measured reflections
1603 independent reflections
1439 reflections with *I* > 2σ(*I*)
*R*_int_ = 0.012

#### Refinement

*R*[*F* ^2^ > 2σ(*F* ^2^)] = 0.037
*wR*(*F* ^2^) = 0.107
*S* = 1.02
1603 reflections
124 parameters
2 restraints
H atoms treated by a mixture of independent and constrained refinement
Δρ_max_ = 0.39 e Å^−3^
Δρ_min_ = −0.39 e Å^−3^

### 

Data collection: *CrysAlis CCD* (Oxford Diffraction, 2009 ▶); cell refinement: *CrysAlis RED* (Oxford Diffraction, 2009 ▶); data reduction: *CrysAlis RED*; program(s) used to solve structure: *SHELXS97* (Sheldrick, 2008 ▶); program(s) used to refine structure: *SHELXL97* (Sheldrick, 2008 ▶); molecular graphics: *PLATON* (Spek, 2009 ▶); software used to prepare material for publication: *SHELXL97*.

## Supplementary Material

Crystal structure: contains datablocks I, global. DOI: 10.1107/S1600536811010518/bt5496sup1.cif

Structure factors: contains datablocks I. DOI: 10.1107/S1600536811010518/bt5496Isup2.hkl

Additional supplementary materials:  crystallographic information; 3D view; checkCIF report

## Figures and Tables

**Table 1:** Hydrogen-bond geometry (Å, °)

*D*—H⋯*A*	*D*—H	H⋯*A*	*D*⋯*A*	*D*—H⋯*A*
N1—H11*N*⋯O1^i^	0.88 (2)	1.85 (2)	2.730 (2)	176 (2)
N1—H12*N*⋯O2^ii^	0.89 (2)	1.88 (2)	2.753 (3)	170 (3)

Symmetry codes: (i) 
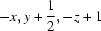
; (ii) 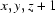.

**Fig. 1.**
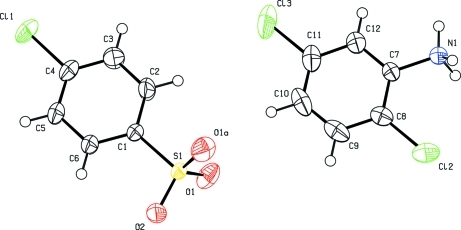
Molecular structure of the title compound, showing the atom labelling scheme. Displacement ellipsoids are drawn at the 50% probability level.

**Fig. 2.**
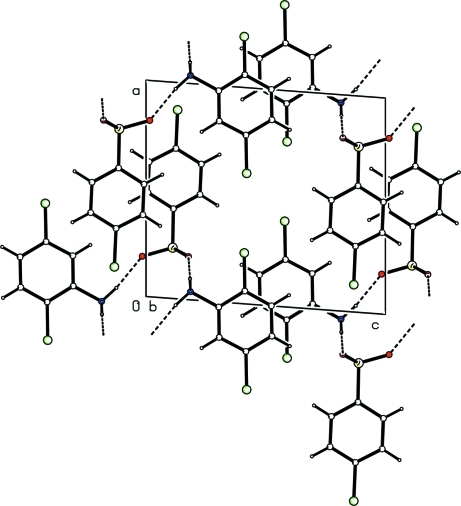
Molecular packing in the title compound. Hydrogen bonds are shown as dashed lines.

## supplementary crystallographic information

### Comment

The amine and sulfonate moieties are important constituents of many important compounds. As a part of studying the substituent effects on the structures of this class of compounds (Gowda *et al.*, 2004*a*, 2004*b*, 2007), in the present work, the crystal structure of 2,5-dichloroanilinium, 4-chlorobenzenesulfonate (I) has been determined (Fig. 1). The title compound showed interesting H-bonding in its crystal structure (Fig. 2). It forms the structure through N—H···O(S) hydrogen bonding. Three H-atoms of the positively charged NH_3_ group have three O atoms of the negatively charged sulfonate anion as acceptors, with each oxygen forming H-bonding with three H-atoms, one each from three positively charged NH_3_ groups.

The crystal packing of (I) through N1—H11N···O1, N1—H11aN···O1a and N1—H12N···O2 hydrogen bonding (Table 1) is shown in Fig.2.

### Experimental

The solution of chlorobenzene (10 ml) in chloroform (40 ml) was treated dropwise with chlorosulfonic acid (25 ml) at 0 ° C. After the initial evolution of hydrogen chloride subsided, the reaction mixture was brought to room temperature and poured into crushed ice in a beaker. The chloroform layer was separated, washed with cold water and allowed to evaporate slowly. The residual 4-chlorobenzenesulfonylchloride was treated with 2,5-dichloroaniline in the stoichiometric ratio and boiled for ten minutes. The reaction mixture was then cooled to room temperature and added to ice cold water (100 ml). The resultant title compound (I) was filtered under suction and washed thoroughly with cold water. It was then recrystallized to constant melting point from dilute ethanol.

Prism like colorless single crystals used in X-ray diffraction studies were grown in ethanolic solution by slow evaporation at room temperature.

### Refinement

The N bounded H atoms were located in a difference map and later restrained to the distance N—H = 0.86 (2) Å. The other H atoms were positioned with idealized geometry using a riding model with C—H = 0.93 Å. All H atoms were refined with isotropic displacement parameters set to 1.2 times of the *U*_eq_ of the parent atom.

### Crystal data

**Table:**

C_6_H_6_Cl_2_N^+^·C_6_H_4_ClO_3_S^−^	*F*(000) = 360
*M**_r_* = 354.62	*D*_x_ = 1.630 Mg m^−^^3^
Monoclinic, *P*2_1_/*m*	Mo *K*α radiation, λ = 0.71073 Å
Hall symbol: -P 2yb	Cell parameters from 1811 reflections
*a* = 9.792 (1) Å	θ = 2.7–27.8°
*b* = 6.802 (1) Å	µ = 0.78 mm^−^^1^
*c* = 10.879 (1) Å	*T* = 293 K
β = 94.26 (1)°	Prism, colourless
*V* = 722.60 (15) Å^3^	0.40 × 0.34 × 0.24 mm
*Z* = 2	

### Data collection

**Table:**

Oxford Diffraction Xcalibur diffractometer with a Sapphire CCD detector	1603 independent reflections
Radiation source: fine-focus sealed tube	1439 reflections with *I* > 2σ(*I*)
graphite	*R*_int_ = 0.012
Rotation method data acquisition using ω scans	θ_max_ = 26.4°, θ_min_ = 2.7°
Absorption correction: multi-scan (*CrysAlis RED*; Oxford Diffraction, 2009)	*h* = −12→12
*T*_min_ = 0.745, *T*_max_ = 0.834	*k* = −8→6
2700 measured reflections	*l* = −13→10

### Refinement

**Table:**

Refinement on *F*^2^	Secondary atom site location: difference Fourier map
Least-squares matrix: full	Hydrogen site location: inferred from neighbouring sites
*R*[*F*^2^ > 2σ(*F*^2^)] = 0.037	H atoms treated by a mixture of independent and constrained refinement
*wR*(*F*^2^) = 0.107	*w* = 1/[σ^2^(*F*_o_^2^) + (0.0626*P*)^2^ + 0.4408*P*] where *P* = (*F*_o_^2^ + 2*F*_c_^2^)/3
*S* = 1.02	(Δ/σ)_max_ = 0.023
1603 reflections	Δρ_max_ = 0.39 e Å^−^^3^
124 parameters	Δρ_min_ = −0.39 e Å^−^^3^
2 restraints	Extinction correction: *SHELXL97* (Sheldrick, 2008), Fc^*^=kFc[1+0.001xFc^2^λ^3^/sin(2θ)]^-1/4^
Primary atom site location: structure-invariant direct methods	Extinction coefficient: 0.036 (4)

### Special details

**Table:**

Experimental. CrysAlis RED (Oxford Diffraction, 2009) Empirical absorption correction using spherical harmonics, implemented in SCALE3 ABSPACK scaling algorithm.
Geometry. All e.s.d.'s (except the e.s.d. in the dihedral angle between two l.s. planes) are estimated using the full covariance matrix. The cell e.s.d.'s are taken into account individually in the estimation of e.s.d.'s in distances, angles and torsion angles; correlations between e.s.d.'s in cell parameters are only used when they are defined by crystal symmetry. An approximate (isotropic) treatment of cell e.s.d.'s is used for estimating e.s.d.'s involving l.s. planes.
Refinement. Refinement of *F*^2^ against ALL reflections. The weighted *R*-factor *wR* and goodness of fit *S* are based on *F*^2^, conventional *R*-factors *R* are based on *F*, with *F* set to zero for negative *F*^2^. The threshold expression of *F*^2^ > σ(*F*^2^) is used only for calculating *R*-factors(gt) *etc*. and is not relevant to the choice of reflections for refinement. *R*-factors based on *F*^2^ are statistically about twice as large as those based on *F*, and *R*- factors based on ALL data will be even larger.

### Fractional atomic coordinates and isotropic or equivalent isotropic displacement parameters (Å^2^)

**Table:**

	*x*	*y*	*z*	*U*_iso_*/*U*_eq_	
Cl1	0.87369 (7)	0.7500	0.13209 (9)	0.0577 (3)	
S1	0.23468 (6)	0.7500	0.11153 (5)	0.0274 (2)	
O1	0.19906 (14)	0.5759 (2)	0.17720 (16)	0.0519 (4)	
O2	0.1854 (2)	0.7500	−0.01564 (19)	0.0575 (7)	
C1	0.4152 (2)	0.7500	0.1146 (2)	0.0277 (5)	
C2	0.4901 (3)	0.7500	0.2257 (3)	0.0740 (15)	
H2	0.4454	0.7500	0.2982	0.089*	
C3	0.6311 (3)	0.7500	0.2313 (3)	0.0833 (17)	
H3	0.6818	0.7500	0.3071	0.100*	
C4	0.6952 (3)	0.7500	0.1251 (3)	0.0398 (7)	
C5	0.6231 (3)	0.7500	0.0130 (3)	0.0406 (7)	
H5	0.6684	0.7500	−0.0592	0.049*	
C6	0.4811 (3)	0.7500	0.0082 (3)	0.0372 (6)	
H6	0.4307	0.7500	−0.0677	0.045*	
Cl2	−0.23538 (8)	0.7500	0.58953 (8)	0.0540 (3)	
Cl3	0.39589 (11)	0.7500	0.57932 (12)	0.0947 (5)	
N1	−0.0373 (2)	0.7500	0.8143 (2)	0.0293 (5)	
H11N	−0.0933 (19)	0.852 (3)	0.8172 (19)	0.035*	
H12N	0.027 (3)	0.7500	0.876 (2)	0.035*	
C7	0.0232 (3)	0.7500	0.6963 (2)	0.0298 (5)	
C8	−0.0594 (3)	0.7500	0.5875 (3)	0.0365 (6)	
C9	0.0000 (4)	0.7500	0.4767 (3)	0.0487 (8)	
H9	−0.0552	0.7500	0.4033	0.058*	
C10	0.1392 (4)	0.7500	0.4734 (3)	0.0530 (9)	
H10	0.1790	0.7500	0.3984	0.064*	
C11	0.2195 (4)	0.7500	0.5822 (3)	0.0504 (8)	
C12	0.1633 (3)	0.7500	0.6941 (3)	0.0425 (7)	
H12	0.2190	0.7500	0.7672	0.051*	

### Atomic displacement parameters (Å^2^)

**Table:**

	*U*^11^	*U*^22^	*U*^33^	*U*^12^	*U*^13^	*U*^23^
Cl1	0.0194 (3)	0.0852 (7)	0.0683 (6)	0.000	0.0028 (3)	0.000
S1	0.0183 (3)	0.0362 (4)	0.0279 (3)	0.000	0.0028 (2)	0.000
O1	0.0376 (8)	0.0493 (9)	0.0692 (10)	−0.0112 (7)	0.0078 (7)	0.0157 (8)
O2	0.0271 (10)	0.114 (2)	0.0306 (10)	0.000	−0.0029 (8)	0.000
C1	0.0217 (11)	0.0327 (13)	0.0290 (12)	0.000	0.0029 (9)	0.000
C2	0.0256 (15)	0.167 (5)	0.0293 (14)	0.000	0.0043 (12)	0.000
C3	0.0268 (15)	0.187 (5)	0.0349 (16)	0.000	−0.0058 (13)	0.000
C4	0.0198 (12)	0.0510 (17)	0.0487 (16)	0.000	0.0037 (11)	0.000
C5	0.0269 (13)	0.0555 (18)	0.0406 (15)	0.000	0.0113 (11)	0.000
C6	0.0257 (13)	0.0556 (17)	0.0304 (12)	0.000	0.0033 (10)	0.000
Cl2	0.0422 (4)	0.0676 (6)	0.0495 (5)	0.000	−0.0150 (3)	0.000
Cl3	0.0499 (6)	0.1572 (13)	0.0820 (7)	0.000	0.0370 (5)	0.000
N1	0.0267 (10)	0.0344 (12)	0.0268 (10)	0.000	0.0009 (8)	0.000
C7	0.0338 (13)	0.0290 (13)	0.0267 (12)	0.000	0.0040 (10)	0.000
C8	0.0450 (16)	0.0314 (14)	0.0323 (13)	0.000	−0.0029 (11)	0.000
C9	0.072 (2)	0.0452 (17)	0.0279 (14)	0.000	−0.0037 (14)	0.000
C10	0.078 (2)	0.0502 (19)	0.0336 (15)	0.000	0.0208 (15)	0.000
C11	0.0479 (18)	0.057 (2)	0.0484 (17)	0.000	0.0202 (14)	0.000
C12	0.0368 (15)	0.0563 (19)	0.0348 (14)	0.000	0.0054 (11)	0.000

### Geometric parameters (Å, °)

**Table:**

Cl1—C4	1.744 (3)	Cl2—C8	1.725 (3)
S1—O2	1.431 (2)	Cl3—C11	1.730 (4)
S1—O1^i^	1.4390 (16)	N1—C7	1.453 (3)
S1—O1	1.4390 (16)	N1—H11N	0.884 (15)
S1—C1	1.766 (2)	N1—H12N	0.886 (18)
C1—C6	1.367 (4)	C7—C12	1.373 (4)
C1—C2	1.366 (4)	C7—C8	1.383 (4)
C2—C3	1.378 (4)	C8—C9	1.377 (4)
C2—H2	0.9300	C9—C10	1.367 (5)
C3—C4	1.355 (5)	C9—H9	0.9300
C3—H3	0.9300	C10—C11	1.371 (5)
C4—C5	1.363 (4)	C10—H10	0.9300
C5—C6	1.387 (4)	C11—C12	1.374 (4)
C5—H5	0.9300	C12—H12	0.9300
C6—H6	0.9300		
			
O2—S1—O1^i^	113.77 (8)	C5—C6—H6	119.9
O2—S1—O1	113.77 (8)	C7—N1—H11N	109.0 (14)
O1^i^—S1—O1	110.72 (14)	C7—N1—H12N	111 (2)
O2—S1—C1	106.49 (12)	H11N—N1—H12N	112.4 (18)
O1^i^—S1—C1	105.65 (8)	C12—C7—C8	120.5 (3)
O1—S1—C1	105.65 (8)	C12—C7—N1	119.2 (2)
C6—C1—C2	119.6 (2)	C8—C7—N1	120.3 (2)
C6—C1—S1	121.2 (2)	C9—C8—C7	119.4 (3)
C2—C1—S1	119.2 (2)	C9—C8—Cl2	119.9 (2)
C1—C2—C3	120.6 (3)	C7—C8—Cl2	120.7 (2)
C1—C2—H2	119.7	C10—C9—C8	120.6 (3)
C3—C2—H2	119.7	C10—C9—H9	119.7
C4—C3—C2	119.2 (3)	C8—C9—H9	119.7
C4—C3—H3	120.4	C11—C10—C9	119.1 (3)
C2—C3—H3	120.4	C11—C10—H10	120.4
C3—C4—C5	121.4 (3)	C9—C10—H10	120.4
C3—C4—Cl1	119.3 (2)	C10—C11—C12	121.6 (3)
C5—C4—Cl1	119.3 (2)	C10—C11—Cl3	119.6 (3)
C4—C5—C6	119.0 (3)	C12—C11—Cl3	118.9 (3)
C4—C5—H5	120.5	C11—C12—C7	118.8 (3)
C6—C5—H5	120.5	C11—C12—H12	120.6
C1—C6—C5	120.2 (3)	C7—C12—H12	120.6
C1—C6—H6	119.9		
			
O2—S1—C1—C6	0.0	C4—C5—C6—C1	0.0
O1^i^—S1—C1—C6	−121.30 (8)	C12—C7—C8—C9	0.000 (1)
O1—S1—C1—C6	121.30 (8)	N1—C7—C8—C9	180.0
O2—S1—C1—C2	180.0	C12—C7—C8—Cl2	180.0
O1^i^—S1—C1—C2	58.70 (8)	N1—C7—C8—Cl2	0.0
O1—S1—C1—C2	−58.70 (8)	C7—C8—C9—C10	0.000 (1)
C6—C1—C2—C3	0.0	Cl2—C8—C9—C10	180.0
S1—C1—C2—C3	180.0	C8—C9—C10—C11	0.0
C1—C2—C3—C4	0.0	C9—C10—C11—C12	0.000 (1)
C2—C3—C4—C5	0.0	C9—C10—C11—Cl3	180.0
C2—C3—C4—Cl1	180.0	C10—C11—C12—C7	0.000 (1)
C3—C4—C5—C6	0.0	Cl3—C11—C12—C7	180.0
Cl1—C4—C5—C6	180.0	C8—C7—C12—C11	0.0
C2—C1—C6—C5	0.0	N1—C7—C12—C11	180.0
S1—C1—C6—C5	180.0		

Symmetry codes: (i) *x*, −*y*+3/2, *z*.

### Hydrogen-bond geometry (Å, °)

**Table:**

*D*—H···*A*	*D*—H	H···*A*	*D*···*A*	*D*—H···*A*
N1—H11N···O1^ii^	0.88 (2)	1.85 (2)	2.730 (2)	176 (2)
N1—H12N···O2^iii^	0.89 (2)	1.88 (2)	2.753 (3)	170 (3)

Symmetry codes: (ii) −*x*, *y*+1/2, −*z*+1; (iii) *x*, *y*, *z*+1.

## References

[bb1] Gowda, B. T., Foro, S. & Fuess, H. (2007). *Acta Cryst.* E**63**, o2570.

[bb2] Gowda, B. T. & Shetty, M. (2004*a*). *J. Phys. Org. Chem.* **17**, 848–864.

[bb3] Gowda, B. T., Svoboda, I. & Fuess, H. (2004*b*). *Z. Naturforsch. Teil A*, **55**, 845–852.

[bb4] Oxford Diffraction (2009). *CrysAlis CCD* and *CrysAlis RED* Oxford Diffraction Ltd, Yarnton, England.

[bb5] Sheldrick, G. M. (2008). *Acta Cryst.* A**64**, 112–122. 10.1107/S0108767307043930 18156677

[bb6] Spek, A. L. (2009). *Acta Cryst.* D**65**, 148–155. 10.1107/S090744490804362X PMC2631630 19171970

